# Visceral adiposity syndrome

**DOI:** 10.1186/s13098-016-0156-2

**Published:** 2016-07-19

**Authors:** Heno F. Lopes, Maria Lúcia Corrêa-Giannella, Fernanda M. Consolim-Colombo, Brent M. Egan

**Affiliations:** Universidade Nove de Julho-UNINOVE, Rua Vergueiro 235/249, 2 subsolo, Liberdade, São Paulo, CEP: 01504-001 Brazil; Instituto do Coração do Hospital das Clinicas da Faculdade de Medicina da Universidade de São Paulo, São Paulo, Brazil; Laboratório de Investigação Médica (LIM-18) e Centro de Terapia Celular e Molecular (NUCEL/NETCEM) da Faculdade de Medicina da Universidade de São Paulo, São Paulo, SP Brazil; Greenville Health System and Department of Medicine, Care Coordination Institute, University of South Carolina-Greenville, Greenville, SC USA

**Keywords:** Insulin resistance, Adipocytokines, Adipose tissue, Sympathetic activity, Parasympathetic activity, Visceral obesity syndrome

## Abstract

The association of anthropometric (waist circumference) and hemodynamic (blood pressure) changes with abnormalities in glucose and lipid metabolism has been motivation for a lot of discussions in the last 30 years. Nowadays, blood pressure, body mass index/abdominal circumference, glycemia, triglyceridemia, and HDL-cholesterol concentrations are considered in the definition of Metabolic syndrome, referred as Visceral adiposity syndrome (VAS) in the present review. However, more than 250 years ago an association between visceral and mediastinal obesity with hypertension, gout, and obstructive apnea had already been recognized. Expansion of visceral adipose tissue secondary to chronic over-consumption of calories stimulates the recruitment of macrophages, which assume an inflammatory phenotype and produce cytokines that directly interfere with insulin signaling, resulting in insulin resistance. In turn, insulin resistance (IR) manifests itself in various tissues, contributing to the overall phenotype of VAS. For example, in white adipose tissue, IR results in lipolysis, increased free fatty acids release and worsening of inflammation, since fatty acids can bind to Toll-like receptors. In the liver, IR results in increased hepatic glucose production, contributing to hyperglycemia; in the vascular endothelium and kidney, IR results in vasoconstriction, sodium retention and, consequently, arterial hypertension. Other players have been recognized in the development of VAS, such as genetic predisposition, epigenetic factors associated with exposure to an unfavourable intrauterine environment and the gut microbiota. More recently, experimental and clinical studies have shown the autonomic nervous system participates in modulating visceral adipose tissue. The sympathetic nervous system is related to adipose tissue function and differentiation through beta_1_, beta_2_, beta_3_, alpha_1_, and alpha_2_ adrenergic receptors. The relation is bidirectional: sympathetic denervation of adipose tissue blocks lipolysis to a variety of lipolytic stimuli and adipose tissue send inputs to the brain. An imbalance of sympathetic/parasympathetic and alpha_2_ adrenergic/beta_3_ receptor is related to visceral adipose tissue storage and insulin sensitivity. Thus, in addition to the well-known factors classically associated with VAS, abnormal autonomic activity also emerges as an important factor regulating white adipose tissue, which highlights complex role of adipose tissue in the VAS.

## Background

As the global epidemic of obesity that emerged in the latter part of the 20th century A.D. continues into the 21st century, a syndrome comprised of risk factors for cardiovascular disease has growing health and economic implications worldwide. This syndrome has various names, of which the best known is probably Metabolic Syndrome. A key driver of the obesity epidemic and related cardiometabolic complications is ‘access to excess’ calories, sugar, fat, salt, labor saving devices and passive entertainment. And, while the epidemic had its roots in developed economies ~40 years ago, during the past 10–20 years it has firmly established residence in most emerging economies. In addition to increasingly prevalent overweight and obesity, mean age is also increasing as birth rates slow in many nations, which further drives this condition. To further amplify risk, in the United States and Europe, the Euro-Caucasian population is growing proportionately less than other racial-ethnic groups, which are typically more susceptible to the ‘diabetogenic’ effects of ‘access to excess’. These observations provide evidence for a syndrome driven by a progressively better but incompletely defined interplay of genetic, environmental, and developmental factors.

Given the constellation and complex interaction of factors underlying the development of Metabolic Syndrome, this is an especially opportune time to review its history and summarize an array of important physiopathologic insights. It is our intent that this information will stimulate progress in preventing and managing this condition and further scientific inquiry to lessen its adverse global health and economic impact.

## Historical aspects

More than 250 years ago, JB Morgagni described an association between visceral and mediastinal obesity, hypertension, gout, and obstructive sleep apnea [1]. He used only a knife for anatomical dissection and his skills to provide the first observations about a cluster of cardiovascular risk factors that we will refer as Visceral Adiposity Syndrome (VAS).

The next three landmarks reports were published in the 1920s. First, two physicians from Vienna, Karl Hitzenberger, and Martin Richter–Quittner made clinical observations during World War I that led to two papers on metabolic factors, hypertension, diabetes and vascular disease [2, 3]. Second, Eskil Kylin, a Swedish physician, described an association between hypertension, hyperglycemia, obesity, and hyperuricemia [4].

Third, William Preble, a Boston physician, made observations from 1000 patients and insurance data, which led him to conclude that “overweight of 15 or more pounds is an increasingly serious condition with advancing years, conducive to heart, arterial and kidney disease, diabetes and hypertension”. He reported that albuminuria was common in overweight patients and documented they were more likely to die from diabetes, heart disease, other circulatory diseases and kidney disease. He also observed that weight loss reduced blood pressure and albuminuria [5]. Preble concluded, “…It is the duty of the physician to acquaint his patient and the community with the gravity of the condition (overweight and obesity)…”.

Insulin resistance, an important mechanism for the VAS was considered as a risk factor for diabetes in the 1930s by Himsworth [6–8]. In the 1940s and 1950s, Jean Vague, a French physician, described a strong relationship between android fat distribution and type 2 diabetes, atherosclerosis, and gout [9, 10]. In the 1960s, Albrink and Meigs described relationships between obesity (skinfold thickness), dyslipidemia and dysglycemia [11]. Moreover, Avogaro and Crepaldi described the association between hyperlipidemia, obesity, hypertension, diabetes and coronary artery disease, which they called the Plurimetabolic Syndrome [12]. In the late 1970s, German investigators [13, 14] described the association between obesity, hypertension, hyperlipidemia, and diabetes and originated the term Metabolic Ssyndrome.

Additional important observations followed in the late 1980s and early 1990s. Reaven described Syndrome X and called attention to insulin resistance as a central feature linking diabetes and impaired glucose tolerance, elevated triglycerides and reduced HDL-cholesterol, and hypertension [15]. Kaplan then described the Deadly Quartet comprised of upper body obesity, glucose intolerance, hypertriglyceridemia and hypertension [16]. Ferrannini and colleagues discussed the same cluster of cardiovascular risk factors and found the term Insulin resistance syndrome more appropriate [17].

In 1985, Ohlson et al. reported that body fat distribution was an important risk factor for diabetes after a follow-up of 13.5 years [18]. In a prospective cohort study, body fat distribution was related to cardiovascular risk in women [19]. Several subsequent studies, summarized in reviews and meta-analyses, found that VAS was related to increased risk for cardiovascular disease [20], chronic kidney disease [21], and several types of cancer [22].

In 1998 [23] the World Health Organization was the first organization to provide a unified definition of Metabolic Syndrome (Table 1) with diabetes or impaired glucose tolerance (IGT) as major clinical features. In patients without diabetes or IGT, glucose uptake in the lowest quartile during a euglycemic hyperinsulinemic clamp also sufficed as evidence for insulin resistance. Individuals required, at least, two other clinical features including hypertension, hypertriglyceridemia, low-HDL, obesity or elevated waist: hip ratio or microalbuminuria.

**Table 1:** Metabolic syndrome definitions provided by various authorities

Authority, yr	WHO 1998	EGIR 1999	ATP III (2001/4)	IDF 2005
Insulin resistance	IGT, IFG, T2D; insulin resistance + 2 below	Plasma insulin >75th percentile + 2 below	None; any 3 of the 5 below	None
BMI or body fat distribution	Men WHR >0.90 Women WHR >0.85 or BMI >30	WC ≥94 cm men or ≥80 cm women	WC >102 cm men or >88 cm women	Increased WC (pop. specific) + 2 below
Lipids	TG ≥150 mg/dL HDL <35 men or <39 women	TG ≥ 150 mg/dL HDL <39 in men or women	TG ≥150 mg/dL HDL <40 men or <50 women	TG ≥150 mg/dL or Rx, HDL <40 men or <50 women or Rx
Glucose	IGT, IFG, T2D	IGT or IFG	≥110 mg/dL	≥100 mg/dL or diabetes
Blood pressure	≥140/≥90	≥140/≥90 or Htn Rx	≥130/≥85	≥130/≥85 or Htn Rx
Other	Urine alb >20 mg /min; ACR >30 mg/g			

*WHO* World Health Organization, *EGIR* European Group for the study of Insulin resistance, *ATP* adult treatment panel, *IDF* international diabetes federation, *IGT* impaired glucose tolerance, *IFG* impaired fasting glucose, *T2D* type 2 diabetes, *BMI* body mass index, *WHR* waist:hip ratio, *Htn* hypertension, *Rx* pharmacologic treatment, *alb* albumin, *ACR* albumin:creatinine ratio

In 1999, The European Group for the Study of Insulin Resistance [24] proposed that three of five clinical criteria were sufficient to define the Metabolic Syndrome, including insulin resistance and two or more of central obesity, high triglycerides or low HDL, hypertension, and fasting glucose ≥6.1 mmol/L (Table 1). In 2001 [25], the National Cholesterol Education/Adult Treatment Panel III proposed that Metabolic syndrome was defined by three or more of waist circumference >40 inches (102 cm) in men or >35 inches (88 cm) in women, fasting glucose ≥110 mg/dL (6.1 mmol/L), subsequently revised to ≥100 mg/dL (5.6 mmol/L) or treatment for diabetes, blood pressure ≥130 mmHg systolic or ≥85 mmHg diastolic or treatment for hypertension, triglycerides ≥150 mg/dL (1.7 mmol/L), or HDL <50 (1.3 mmol/L) for women or <40 mg/dL (1.0 mmol/L) for men. The International Diabetes Federation [26] adopted waist circumference, defined with ethinicity specific values, as a *sine qua non* criteria for the Metabolic Syndrome diagnosis.

The subsequent sections summarize concepts and research which have highlighted various important facets of the VAS following these seminal reports.

## Insulin resistance and tissue inflammation

Insulin resistance is defined as the reduction of the metabolic (but not mitogenic) effects of insulin following its binding to the insulin receptor. Insulin resistance involves impaired activation of insulin substrate 1 (IRS1) and, subsequently, of the phosphatidylinositol 3-kinase (PI3K) pathway in insulin-sensitive tissues [27]. Visceral adiposity is strongly associated with insulin resistance, but genetic and other factors also predispose to the development of this condition, since there are obese patients without insulin resistance as well as lean individuals who are resistant [28]. An interesting point to consider is that individuals who have subnormal amounts of adipose tissue, such as the ones presenting monogenic lipodystrophy, also develop severe IR. This fact corroborates the hypothesis that adipose tissue failure (loss of capacity of expanding to store excess calories) is more important in the pathogenesis of VAS than the degree of obesity [29].

Several complex and interwoven mechanisms underlie IR in the presence of visceral adiposity, and some of them will be shortly addressed. The first question to be answered is why visceral fat? It is well established that visceral and subcutaneous white adipose tissues (WAT) are different concerning adipocyte size and metabolic activity [30]. This includes the ability to respond to the antilipolytic effects of insulin and to the lipolytic effects of catecholamines, which are, respectively, lower and higher in visceral than in subcutaneous tissue [31], featuring visceral WAT as more pathogenic [30].

Chronic over-consumption of nutrients concomitantly with insufficient energy expenditure exceed the storage capacity of glycogen and triglycerides by the “professional” metabolic tissues, namely, liver, muscle and WAT. In such situation, other tissues are exposed to supraphysiological concentrations of these nutrients, where they exert deleterious effects [32].

Saturated fatty acids, for instance, can bind to receptors that participate in pathogen recognition and innate immunity, the Toll-like receptors (TLR) 2 and 4, especially in adipocytes and macrophages. The consequent induction of c-Jun N-terminal kinase (JNK) results in phosphorylation of IRS1 in the amino acids serine (instead of tyrosine, which is phosphorylated under physiological conditions), decreasing insulin receptor signaling. The activation of the aforementioned pro-inflammatory pathway also stimulates the transcription factor nuclear factor kappa B (NFkB), resulting in the production of inflammatory cytokines such as tumor necrosis factor alpha (TNFα) and interleukin (IL) 6 [33]. These cytokines amplify the JNK signaling pathway and further contribute to impaired signaling through the insulin receptor [34].

Simultaneously, the expansion of WAT secondary to increased caloric intake leads to necrosis/apoptosis of hypertrophic adipocytes and release of large fat droplets. The fat droplets are toxic to the surrounding cells and eventually stimulate the recruitment of bone marrow macrophages, leading to a significant increase of these cells in the WAT of obese subjects. These macrophages, probably stimulated by cellular debris, assume an inflammatory phenotype, also known as classic or M1, characterized by the expression of proinflammatory cytokines (TNFα, IL1 and IL6), which aggravate insulin resistance. M1 macrophages differ from the macrophages that reside in the WAT of lean individuals, which present a phenotype known as M2 or alternative, characterized by the expression of anti-inflammatory cytokines such as IL10 and transforming growth factor-beta (TGFβ) [32].

The activation of the JNK pathway by TNFα and IL6 together with the oxidative stress triggered by the excess of nutrients downregulate the expression of adiponectin (and their receptors), a hormone produced by adipocytes. Adiponectin exerts anti-inflammatory effects, increases transport and uptake of fatty acids by muscles, stimulates glucose uptake by muscle and WAT and decreases hepatic glucose production. Therefore, low adiponectin concentrations contribute to impaired glucose homeostasis and insulin resistance [35].

Insulin resistance manifests itself differentially in various tissues, which contributes to the overall phenotype of VAS. For example, insulin resistance in: (1) WAT, results in lipolysis, increasing free fatty acids into the circulation, which exacerbate the deleterious cycle of hyperlipidemia—inflammation—insulin resistance; (2) the liver, results in increased hepatic glucose production (via glycogenolysis and gluconeogenesis), contributing to hyperglycemia [32]. Hepatic insulin resistance also stimulates the synthesis of hepatocyte growth factor (HGF) [36] and betatrophin [37], growth factors produced by the liver (and also by the fat tissue) that stimulate β-cell hyperplasia, which with other factors, such as free fat acids and glucose, contributes to the compensatory hyperinsulinemia that often accompanies and actually worsens IR [38]; (3) skeletal muscle, a tissue with marked metabolic flexibility to consume and store glucose and lipids, results in increased content of free fatty acids and triglycerides [39], maintaining the vicious cycle hyperlipidemia—inflammation—insulin resistance; (4) in the central nervous system (CNS), at least in animal models, results in hyperphagia and increased fat mass [40];(5) in pancreatic β-cells results in diminished inhibition of glucagon secretion by insulin; the consequent hyperglucagonemia increases hepatic glucose production, contributing to hyperglycemia [41]; (6) in pancreatic β-cells results in reduced glucose-stimulated insulin secretion [42] and, in rodents, in lower pancreatic content of insulin [43]; (7) in the vascular endothelium results in vasoconstriction and, consequently, arterial hypertension, since insulin normally induces PI3K-dependent production of nitric oxide and vasodilatation [44]; (8) in the kidney results in anti-natriuresis at several sites along the renal tubule, causing sodium retention [45] as well as in increased circulating concentrations of components of the renin-angiotension-aldosterone system [46], both changes contributing to hypertension.

Insulin resistance in some of the former target sites aggravates glycemic homeostasis, increasing the formation of advanced glycation end products (AGEs). AGEs are derived from non-enzymatic reactions between glucose and proteins, nucleic acid and lipids. Endogenously derived AGEs, as well as exogenously generated AGEs formed during high-heat cooking, can promote inflammation. This happens via receptors that bind to advanced glycation end products, such as RAGE (advanced glycosylation end product-specific receptor), TLR4 and other receptors that regulate the activity of NFκB [47], constituting another link between diet and insulin resistance.

An interesting aspect that deserves attention concerns the effects of compensatory hyperinsulinemia: although it is usually considered a by-product of the insulin resistance, hyperinsulinemia exerts its effects, overstimulating certain pathways of insulin action in various cells [27]. For instance, the expression of Sterol Regulatory Element Binding Protein 1c (SREBP1c), a transcription factor that modulates the expression of lipogenic enzymes, is increased by chronic hyperinsulinemia in the liver of mice exhibiting insulin resistance. As a result, de novo fatty acid biosynthesis is enhanced and contributes to non-alcoholic fatty liver disease (NAFLD) [48], the hepatic component of the VAS. Another potential consequence of prolonged hyperinsulinemia is the exacerbation of inflammation since it has been demonstrated that in vitro, insulin exerts long-term proinflammatory action, by amplifying effects of the cytokine-NFkB axis [49]. An important implication of hyperinsulinemia that needs to be clearly established is whether neoplastic cells in patients with insulin resistance remain sensitive to insulin’s proliferative actions. If so, then hyperinsulinemia could be one of the mechanisms proposed to explain the increased frequency of cancer that accompanies the obesity epidemic [50]. Other mechanisms that may explain the increased cancer risk include the mitogenic effect of insulin through either insulin-like growth factor-1 (IGF1) receptors or through hybrid IGF1-insulin receptors as well as the increased concentrations of free IGF1, as a consequence of the lower concentrations of IGF binding proteins 1 and 2 (IGFBP1 and IGFBP2) in obese individuals [51].

## Other players

### Prenatal reprogramming

During embryonic development, there is significant plasticity, which attempts to adjust the foetal gene expression to the environment, so that the resulting phenotype is adapted to the environmental conditions [52]. Thus, fetuses exposed to suboptimal conditions during intrauterine life (for instance, protein-calorie undernutrition) undergo alterations in gene expression to adjust. However, if the post-natal life provides different conditions from those previously anticipated (for instance, abundance of nutrients), the body will not be prepared for that environment, and is more likely to develop disease [53].

This ability to sustain the changes acquired in intrauterine life in the post-natal life relies on epigenetic mechanisms, namely, post-translational modifications within histones, DNA cytosine methylation and control of gene expression by micro-non-coding RNAs. These molecular processes occur around the DNA, are stable during mitosis and can regulate genome activity regardless of DNA sequence [54]; they allow cells to respond quickly to environmental modifications and “remember” these responses even after removal of the causative stimulus [55].

The Dutch famine has been used to investigate the effects of in utero stress among adults who were born in the western part of The Netherlands at the end of World War II. These individuals were exposed prenatally to undernutrition. The Dutch Famine Cohort study has shown that individuals exposed to maternal undernutrition at any stage of gestation had increased glycaemia as adults. Women exposed to undernutrition at early stages of gestation appeared to be more centrally obese than those not prenatally exposed to famine [56]. Interestingly, higher methylation of genes related to metabolic and cardiovascular diseases, such as the ones encoding leptin, IL10 and ABCA1 were found in individuals prenatally exposed to famine in comparison to their unexposed same-sex siblings [57]. In the same line of investigation, specific associations of low birth weight with VAS were observed in other populations [58, 59], strongly suggesting that, besides environmental and genetic conditions, prenatal reprogramming participates in the susceptibility to the VAS.

### Microbiota

Another player recently recognized as important in the development of VAS is the gut microbiota, comprised of approximately 100 trillion microbes resident in the human intestines, the majority of them belonging to the phyla *Firmicutes* and *Bacteroidetes*. The gut microbiota is influenced by diet composition, which, in turn, influences how food is processed in the gastrointestinal tract [60].

Pathobionts (resident microbes with pathogenic potential) [61] increase by diets enriched in saturated fat, damage the intestinal epithelial cell layer and enable the translocation of one key constituent of many bacteria, lipopolysaccharide (LPS), from the gut lumen into the systemic circulation [62]. LPS molecules bind to TLRs, activating the proinflammatory signaling cascades previously mentioned and eliciting insulin resistance; in the liver, for instance, activation of TLR4 and TLR9 augments TNFα secretion and participates in NAFLD development [60].

On the other hand, a fiber-rich diet exerts beneficial effects on the microbiota composition, decreasing the *Firmicutes*: *Bacteriodetes* ratio and consequently increasing fiber degradation and the production of short-chain fatty acids, such as acetate, propionate, and butyrate [63], which are absorbed in the colon. Acetate and propionate are substrates for lipogenesis and gluconeogenesis while butyrate provides energy for colonic epithelial cells [60]. Additionally, these compounds bind to widely expressed G-protein coupled receptors (GPR41 and GPR43), augmenting energy expenditure, insulin sensitivity, satiety and production of glucagon-like peptide 1 (GLP1) and decreasing inflammation, thereby protecting against VAS [62].

## Autonomic modulation of visceral adipose tissue

The CNS is an important modulator of food intake through different hormonal and neuropeptide signaling pathways [64]. Neuropeptides regulate energy storage in white adipocytes and inhibit brown adipose tissue activation in mammals [65]. Experimental and clinical studies showed a relation between the autonomic nervous system, dietary intake and adipose tissue. Body fat distribution of body fat is a more important risk factor for the development of hypertension and cardiovascular disease than obesity generally. Sympathetic nervous system (SNS) activity contributes to obesity-induced hypertension [66]. Independently of body fat distribution, sympathetic activity seems to be related to different components of the VAS. By stratifying patients with similar obesity degrees according to the presence or absence of high blood pressure, we found a higher surrogate markers of sympathetic activity derived from spectral analysis and greater impairment in several components of Metabolic syndrome in those subjects with high blood pressure [67]. It suggest that high blood pressure carriage in pararel with sympathetic activity in this population. Nonetheless, SNS activity was associated to obesity in obese normotensive subjects [68]. The distribution of body fat seems to be more important to determine the cardiovascular risk than the whole body fat. Alvarez et al. showed a higher sympathetic activity in men with visceral obesity compared to subcutaneous fat levels [69]. Leptin, an important product of visceral adipocytes, is related to increased sympathetic activity, with human obesity-induced hypertension, and associated to a increased termogenic metabolism [70]. The SNS also plays a role in the regulation of mammalian thermogenesis and contributes to changes in energy expenditure that accompany changes in diet. The energy imbalance resulting from metabolic heat in response to cold exposure and to diet intake is covered by the sympathetic nervous system, which suggest an unequivocal relation between sympathetic activity and body fat deposition [71]. Even in healthy subjects body fat showed a direct relationship with SNS activity [72]. Grassi et al. evaluating normal control subjects, subjects with peripheral and central obesity showed a greater sympathetic activity in subjects with central obesity [73]. A recent study analyzed the effects of surgically-induced weight loss in severely obese patients and showed an important reduction in insulin resistance index, leptin levels, and sympathetic activity [74]. Abdominal adiposity loss is associated with diabetogenic and atherogenic markers. In a study evaluating peripheral and central obesity a moderate weight loss (5–10 %) was associated with approximately a 30 % decline of visceral adipose tissue [75]. Different studies suggest a straight relationship between sympathetic activity and central WAT [66, 73]. The relation is bidirectional; sympathetic denervation of WAT blocks lipolysis to a variety of lipolytic stimuli and the use of anterograde transneural viral tracers has defined the sensory input from WAT to the brain [76]. These experiments show the importance of sympathetic innervation in WAT.

The effects of parasympathetic innervation of the adipose tissue are partially elucidated. Kreier et al. used a retrograde transneuronal tracer, i.e., pseudorabies virus, in intra-abdominal fat pads of sympathetically denervated rats to evaluate parasympathetic innervation in adipose tissue [77]. They demonstrated that parasympathetic denervation of WAT significantly reduced insulin-dependent glucose and free fat acid uptake and increased the sensitivity of lipase hormone-sensitive, resulting in an increased intracellular triglyceride breakdown. Thus, evidence suggests that the parasympathetic system is associated with adipogenesis (triglycerides synthesis), whereas the sympathetic system is associated with lipolysis (triglycerides breakdown) in WAT.

SNS modulation of visceral adipose tissue is related to different adrenergic receptors. Adipocyte plasma membranes express beta-1 (β_1_), beta-2 (β_2_), beta-3 (β_3_), alpha-1(α_1_), and alpha-2 (α_2_) adrenergic receptors [78]. The balance between alpha and beta adrenergic receptors emerges as an important variable in regulating adipocyte cell number. Using a murine model with genetically altered balance alpha_2_/beta_3_ adrenoreceptors, Valet et al. demonstrated adipocyte hyperplasia [79]. The fasting state is associated with adipolysis, which is modulated mainly by the β_3_ adrenergic receptor. Conversely, adipogenesis is associated with activation of the parasympathetic system, but the receptor mediating adipogenesis is not well defined [76]. A sympathetic/parasympathetic imbalance as well as an α_2_ adrenergic/β_3_ receptor imbalance are related to visceral adipose tissue storage and insulin sensitivity.

Thus, in addition to the well-known factors classically associated with VAS, the literature suggests an important role for autonomic activity in the regulation of WAT, which highlights the complex role of adipose tissue in the VAS.
